# Anticancer effects of melatonin via regulating lncRNA JPX‐Wnt/β‐catenin signalling pathway in human osteosarcoma cells

**DOI:** 10.1111/jcmm.16894

**Published:** 2021-09-21

**Authors:** Yuan Li, Jilong Zou, Bo Li, Jianyang Du

**Affiliations:** ^1^ Department of Pharmacology, School of Pharmaceutical Sciences, Cheeloo College of Medicine Shandong University Jinan Shandong China; ^2^ Suzhou Research Institute Shandong University Suzhou Jiangsu China; ^3^ Department of Orthopedics the First Affiliated Hospital of Harbin Medical University Harbin Heilongjiang China; ^4^ Department of Orthopedics Sun Yat‐Sen Memorial Hospital of Sun Yat‐Sen University Guangzhou China; ^5^ Department of Neurosurgery Shandong Provincial Hospital Affiliated to Shandong First Medical University Jinan Shandong China

**Keywords:** LncRNAs, melatonin, osteosarcoma, therapeutic methods, Wnt/β‐catenin pathway

## Abstract

Osteosarcoma (OS) is a type of malignant primary bone cancer, which is highly aggressive and occurs more commonly in children and adolescents. Thus, novel potential drugs and therapeutic methods are urgently needed. In the present study, we aimed to elucidate the effects and mechanism of melatonin on OS cells to provide a potential treatment strategy for OS. The cell survival rate, cell viability, proliferation, migration, invasion and metastasis were examined by trypan blue assay, MTT, colony formation, wound healing, transwell invasion and attachment/detachment assay, respectively. The expression of relevant lncRNAs in OS cells was determined by real‐time qPCR analysis. The functional roles of lncRNA JPX in OS cells were further examined by gain and loss of function assays. The protein expression was measured by western blot assay. Melatonin inhibited the cell viability, proliferation, migration, invasion and metastasis of OS cells (Saos‐2, MG63 and U2OS) in a dose‐dependent manner. Melatonin treatment significantly downregulated the expression of lncRNA JPX in Saos‐2, MG63 and U2OS cells. Overexpression of lncRNA JPX into OS cell lines elevated the cell viability and proliferation, which was accompanied by the increased metastasis. We also found that melatonin inhibited the OS progression by suppressing the expression of lncRNA JPX via regulating the Wnt/β‐catenin pathway. Our results suggested that melatonin inhibited the biological functions of OS cells by repressing the expression of lncRNA JPX through regulating the Wnt/β‐catenin signalling pathway, which indicated that melatonin might be applied as a potentially useful and effective natural agent in the treatment of OS.

## 1 INTRODUCTION

Osteosarcoma (OS), characterized by a higher prevalence in adolescents and children, has been regarded as the most common bone malignancy with a high fatality rate.^1^, ^2^ As a highly malignant bone tumour, OS originates from bone marrow mesenchymal stem cells (BMSCs), characterized by the presence of malignant mesenchymal spindle cells and production of malignant osteoid or immature bone.^3^, ^4^ Unfortunately, OS usually occurs in the extremities and easily metastasizes to lung and predominately affects the rapid growth of bones in children and adolescents.^5^ The five‐year survival rate of patients with OS is ~60%–70% and is as low as 20%–28% in patients with metastases.^6^, ^7^ Currently, with the development of various treatment methods for OS, including surgery, radiotherapy and chemotherapy, the rate of metastasis is still about 40%, and the clinical outcome has not been markedly improved.^8^ Thus, a variety of novel agents need to be developed and applied for the treatment of OS as adjuvant therapeutic strategies.

Melatonin (*N*‐acetyl‐5‐methoxytryptamine) is a type of pineal indolamine and naturally occurring derivative of the amino acid tryptophan with diverse biological activities, which is commonly observed in the nature and occurs in unicellular organisms, fungi, plants and animals.^9^, ^10^, ^11^ Melatonin is initially extracted and identified in the bovine pineal tissues. And now, a vast number of studies indicate that this indolamine could be synthesized in the brain, retina, gastrointestinal tract, thymus and skin, and is mainly produced by the pineal gland at night.^12^ Melatonin exerts numerous biological functions, such as sleep induction, biological rhythms modulation, anti‐apoptotic signalling function, vaso‐regulation, anti‐tumour action, antioxidant properties, cytoprotective effects and immunomodulation.^13^, ^14^ According to the extensive involvement of melatonin in various fundamental biological functions, it is anticipated that melatonin possesses beneficial effects in a variety of disorders and diseases, including insomnia, Alzheimer's disease, osteoporosis, Parkinson's disease, fatty liver disease, Huntington's disease, Amyotrophic lateral sclerosis, migraine and headache manifestations and gastrointestinal diseases.^15^, ^16^, ^17^, ^18^, ^19^, ^20^ As an autocoid, a chronobiotic, a sleep‐inducing agent, an immune modulator and a biological adjusting agent, melatonin is also regarded as an anti‐tumour agent, which has significant beneficial roles in the prevention and treatment of diverse cancer types.^21^, ^22^ A series of novel studies clarified the roles of melatonin in multiple cancers due to its anti‐metastatic potential, drug sensitivity restoration, apoptosis induction, growth inhibition and anti‐angiogenic and anti‐invasive actions.^23^, ^24^ However, the functions of melatonin in OS have not been fully clarified and an accurate mechanism of how melatonin properly orchestrates such functions needs to be further investigated.

Long non‐coding RNAs (lncRNAs) are defined as an important kind of the non‐coding RNA family, which is more than 200 nucleotides (nt) in length.^25^, ^26^, ^27^ An increasing number of studies have revealed that lncRNAs participate in various biological processes during life, including transcriptional modification, modulation of chromatin architecture, cellular growth, differentiation, development, RNA processing and cell cycle control.^28^, ^29^, ^30^, ^31^ The relationship between lncRNAs and cancers has been recently revealed. LncRNAs are aberrantly expressed in a variety of cancers, and they have displayed great potential as powerful tumour markers.^32^, ^33^ LncRNA JPX, a molecular switch for X chromosome inactivation, has been demonstrated to play a crucial role in the development of cancers, including lung cancer, hepatocellular carcinoma, oral squamous cell carcinoma, cervical cancer and ovarian cancer.^34^, ^35^, ^36^, ^37^, ^38^, ^39^ Nevertheless, no reports are demonstrating the regulating roles and mechanism of lncRNA JPX in OS.

In the current study, we have investigated the anti‐cancer roles of the melatonin in human OS cell lines and further explored the underlying mechanism. Our results showed that different concentrations of melatonin inhibited cell viability, proliferation, migration and invasion by suppressing the expression of lncRNA JPX. We have also observed that lncRNA JPX was upregulated in OS cells, and lncRNA JPX played a positive role in the biological functions of OS cells. Furthermore, lncRNA JPX exerted its function in OS cells by promoting the Wnt/β‐catenin signalling pathway. Our work presented a novel, natural chemotherapeutic agent that might be applied to treat OS. This study shed the new light on the functions and molecular mechanism of melatonin in the treatment of OS.

## 2 METHODS

### 2.1 Cell lines and cell culture

Human osteoblast cell line hFOB1.1 and human OS cell lines (Saos‐2, MG63 and U2OS) were purchased from American Type Culture Collection (ATCC, USA). Human osteoblast cell line hFOB1.1 was maintained in Dulbecco's modified Eagle's medium (DMEM, Hyclone, USA), which contained 10% foetal bovine serum (FBS) and 1% penicillin‐streptomycin according to ATCC recommendations. Human OS cell lines were cultured in high glucose DMEM (Hyclone, USA) supplemented with 10% FBS and 1% penicillin‐streptomycin. All the cells were cultured in a 37℃, 5% CO_2_ humidified incubator (Thermo, USA). When the density reached 60% confluence, the drug treatment and transfection were carried out.

### 2.2 Drug treatment

Melatonin (>98% purity) was purchased from Sigma, USA. Melatonin stock solution was dissolved in dimethyl sulfoxide (DMSO) following the manufacturer's instruction. For melatonin treatment, the human OS cell lines were incubated in high glucose DMEM containing different concentrations (0.1, 0.5, 1, 1.5 and 2 mM) of melatonin at 37℃ for 48 h. As a comparison, the OS cells from the control group were treated with the same dose of DMSO. Wiki4 was purchased from Chembridge, USA.

### 2.3 Lentiviral transduction

LncRNA JPX‐overexpressing lentivirus, lncRNA JPX‐specific shRNA (shRNA JPX) and scramble negative control (NC) were purchased from GenePharma Co., Ltd. (Shanghai, China). The cells were transfected with lncRNA JPX and shRNA JPX using polybrene (Cyagen Biosciences, USA) according to the manufacturer's instructions. After 48 h, the cells were utilized for additional analysis. The expression level of lncRNA JPX was then confirmed by real‐time qPCR analysis.

### 2.4 Trypan blue assay

In brief, OS cells were seeded into the six‐well plates at a density of 5 × 10^5^ cells/well. After melatonin treatment, the cells were treated with trypsin reagent (Gibco, USA) and the mixture of detached cells was washed using PBS solution twice. Then, the cells were collected and centrifuged at 300 *g* for 10 min. Next, the pellet was resuspended with 500 μl 0.4% trypan blue solution (Thermo, USA). The cells were maintained in the solution for 3 min and then analysed under an automated cell counter (TC10, BioRad). The blue colour represented the dead cells, and the cell survival rate (%) was shown as the percentage of the live cells compared to total cells.

### 2.5 MTT assay

Cell viability was examined using MTT assay. The cells were seeded in a 96‐well (NEST, China) at a density of 5 × 10^4^/ml. Then, the cells were exposed to melatonin or/and lncRNA JPX for 48 h. After 48 h, the medium was discarded and the cells were maintained in 0.5 mg/ml MTT solution at 37℃ temperature for 4 h. Then, the medium was replaced by 150 µl DMSO solution. The optical density (OD) values at a wavelength of 490 nm were detected by using a microplate reader (TECAN, Switzerland).

### 2.6 Colony formation assay

Cell proliferation was detected using colony formation assay. The cells at a density of 5 × 10^2^/ml were plated in six‐well plates (NEST, China) and allowed to grow. The medium was changed every three days. After a 14‐day incubation, the colonies were fixed with the methanol for 15 min and observed by staining with 1% crystal violet (Biosharp, China) for 30 min at room temperature and washed again. At last, the colonies were observed and counted under a microscope (Olympus, Japan).

### 2.7 Wound healing assay

The cell migration was assessed by wound healing assay. The cells were seeded into six‐well plates at a density of 1 × 10^6^/well. The sterile pipette tips were applied to scratch cell layers. After washing with PBS three times to remove the floating cells, the cells were photographed and the wound closing process was observed using an inverted light microscope (Olympus, Japan) at 0 and 48 h, respectively. The images were captured and used for the measurement of the wound width.

### 2.8 Transwell assay

Cell invasion ability was monitored using transwell assay. 24‐well transwell chambers (Corning, USA) and 8‐µm‐sized pore membranes coated with matrigel were used to perform invasion assay. A total of 1 × 10^5^ cells were plated in the upper chamber with 100 μl of serum‐free medium, and the bottom chamber was full of 600 μl normal culture medium. The cells were cultured for 48 h and obtained for transwell assay. The cells in the bottom chamber that had migrated through the membrane were fixed by 4% PFA for 30 min at room temperature and stained with 1% crystal violet for 30 min. At last, the number of invaded cells on the bottom of the membrane was calculated under a microscope.

### 2.9 Attachment and detachment assays

For attachment assay, the cells were plated into 24‐well plates (NEST, China) at a density of 5 × 10^4^ cells/well. After 60 min, the unattached cells were discarded. And the number of attached cells was examined and assessed after trypsinization. The percentage of the attached cells compared to total cells was analysed.

For cell detachment assay, the cells were culture in 24‐well plates at a density of 5 × 10^4^ cells/well. After 24 h, 0.05% trypsin was added into the cells for 3 min to detach the cells. Then, the high glucose DMEM was applied to inactivate the trypsin and the detached cells were collected. The remaining cells were treated with 0.25% trypsin and the cells were counted. The percentage of the detached cells to total cells was obtained.

### 2.10 Analysis of expression by real‐time qPCR analysis

The harvested cells were subjected to total RNA isolation by using TRIzol (Invitrogen, USA) according to the instructions. The quality and purity of RNAs were examined using NanoDrop machine (Thermo, USA). The preparation of the cDNA was conducted following the instructions described in the cDNA synthesis kit manual (Applied Biosystems, USA). The cDNAs were amplified by PCR analysis by using SYBR reagents (Roche, Switzerland). And fold changes were analysed by relative quantification (2^−ΔΔCt^) method. The sequences of primers used for real time qPCR analysis were shown in Table 1.

**TABLE 1:** The primer sequences used for RT‐qPCR analysis

Gene	Primers (5′–3′)
lncRNA LUADT1	Forward: TTTCCGTTCAGCAAATCCACAC
	Reverse: TTAGGTCCAGCACTGTTATCCA
lncRNA ZDHHC8P1	Forward: GAGGTCCTACGCTGTGCTAC
	Reverse: CAAGAAGGACATCTGGGGCG
lncRNA JPX	Forward: TGCAGTCAGAAGGGAGCAAT
	Reverse: CACCGTCATCAGGCTGTCTT
lncRNA LINP1	Forward: TGCCACTGCCATTAGAAGAAC
	Reverse: GCTCACAGAGGAGCTACCCA
lncRNA AGAP2‐AS1	Forward: TACCTTGACCTTGCTGCTCTC
	Reverse: TGTCCCTTAATGACCCCATCC
GAPDH	Forward: TGACGTGCCGCCTGGAAAC
	Reverse: CCGGGCATCGAAGGTGGAAGAG

#### 2.10.1 Protein extraction and western blot

Quantitative analysis on the protein expression level was conducted by western blot analysis according to the previous study.^40^ Briefly, the cell lysates were loaded onto and separated by sodium dodecyl sulphate‐polyacrylamide gel electrophoresis (SDS‐PAGE) and transferred into nitrocellulose membranes (Millipore, USA). The membranes were blocked in 5% non‐fat milk, followed by incubation with primary antibodies. Then, the membranes were incubated with the secondary antibodies (1:1000, Abcam, USA) for 60 min. The primary antibodies for Western blot analysis were as follows: anti‐β‐catenin (ab32572, Abcam, Britain), anti‐MYC (sc‐4084, Santa Cruz Biotechnology, China), Cyclin D1 (ab134175, Abcam, Britain) and Axin2 (2151, Cell Signaling Technology, USA).

### 2.11 Statistical analysis

All the experiments were performed at least three times independently. Data were presented as mean ± SD. Statistical analysis was performed using GraphPad Prism software (Graphpad, USA). Statistical significance was assessed by the Student's *t*‐test or one‐way multivariate analysis of variance (ANOVA). The data conformed to the normal distribution. *p* < 0.05 were considered significant difference.

## 3 RESULTS

### 3.1 Inhibitory effects of melatonin on the cell growth and proliferation of OS cells

**FIGURE 1:**
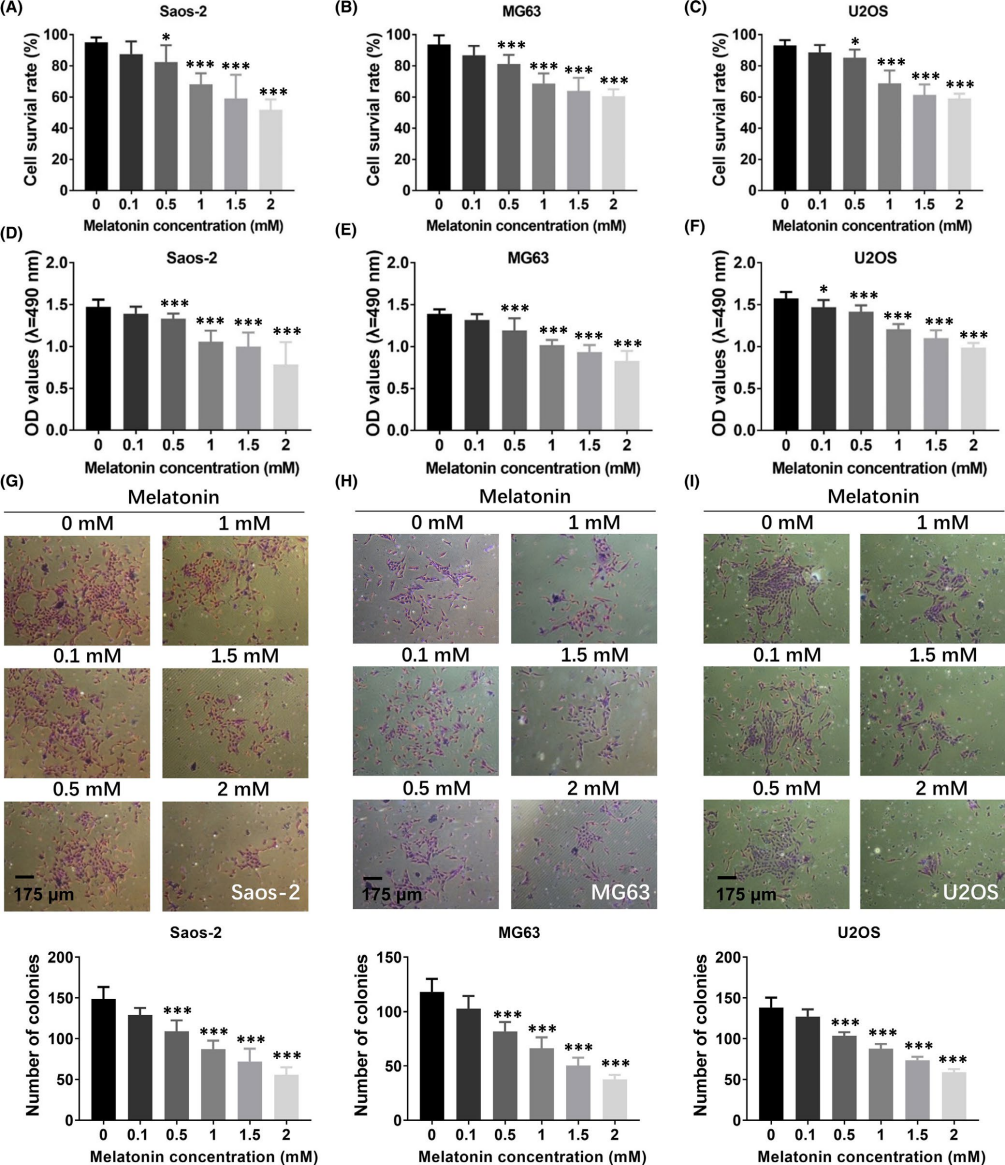
The effects of melatonin on the cell growth and proliferation of OS cells. (A–C) The roles of melatonin in the cell survival of Saos‐2 (A), MG63 (B) and U2OS (C) was measured by trypan blue assay. (D–F) The cell viability in Saos‐2 (D), MG63 (E) and U2OS (F) treated with different concentrations of melatonin was determined by MTT assay, respectively. (G–I) The cell proliferation ability was measured by colony formation assay in Saos‐2 (G), MG63 (H) and U2OS (I) in the presence of melatonin. Scale bar, 175 μm. Significant difference relative to control group was presented as *p < 0.05; **p < 0.01 and ***p < 0.001

Initially, we explored the effects of melatonin on the cell growth of OS cells. Human OS cell lines (Saos‐2, MG63 and U2OS) were treated with melatonin at different concentrations (0.1, 0.5, 1, 1.5 and 2 mM) for 48 h. Trypan blue exclusion assay was performed to detect the effects of melatonin on the cell survival rate of OS cells. As shown in Figure 1A–C, melatonin could decrease the cell survival rate of Saos‐2, MG63 and U2OS cells in a concentration‐dependent manner, suggesting that melatonin treatment induced a gradual increase of dead cells. Among different concentrations of melatonin, the inhibitory effects were markedly observed at the concentration of 1, 1.5 and 2 mM in these three cell lines (Figure 1A–C). We then identified whether melatonin could induce OS cell viability decrease in OS cells, which was examined quantitatively by MTT assay. The results of MTT assay revealed that treatment of OS cells with melatonin at 0.1, 0.5, 1, 1.5 and 2 mM gradually inhibited the cell viability of Saos‐2, MG63 and U2OS cells (Figure 1D–F). Interestingly, melatonin at 1, 1.5 and 2 mM resulted in the significant decrease of cell viability in OS cells (Figure 1D–F). To assess the roles of melatonin in the proliferation of OS cells, three OS cells (Saos‐2, MG63 and U2OS cells) were employed. As presented in Figure 1G–I, the results of colony formation assays showed that the colony number was progressively decreased after melatonin treatment, which declared that the proliferation ability was obviously suppressed in the presence of melatonin. These results confirmed the suppressive effects of melatonin on the cell growth and proliferation in OS cells.

### 3.2 Melatonin induces the reduced metastasis in OS cells

**FIGURE 2:**
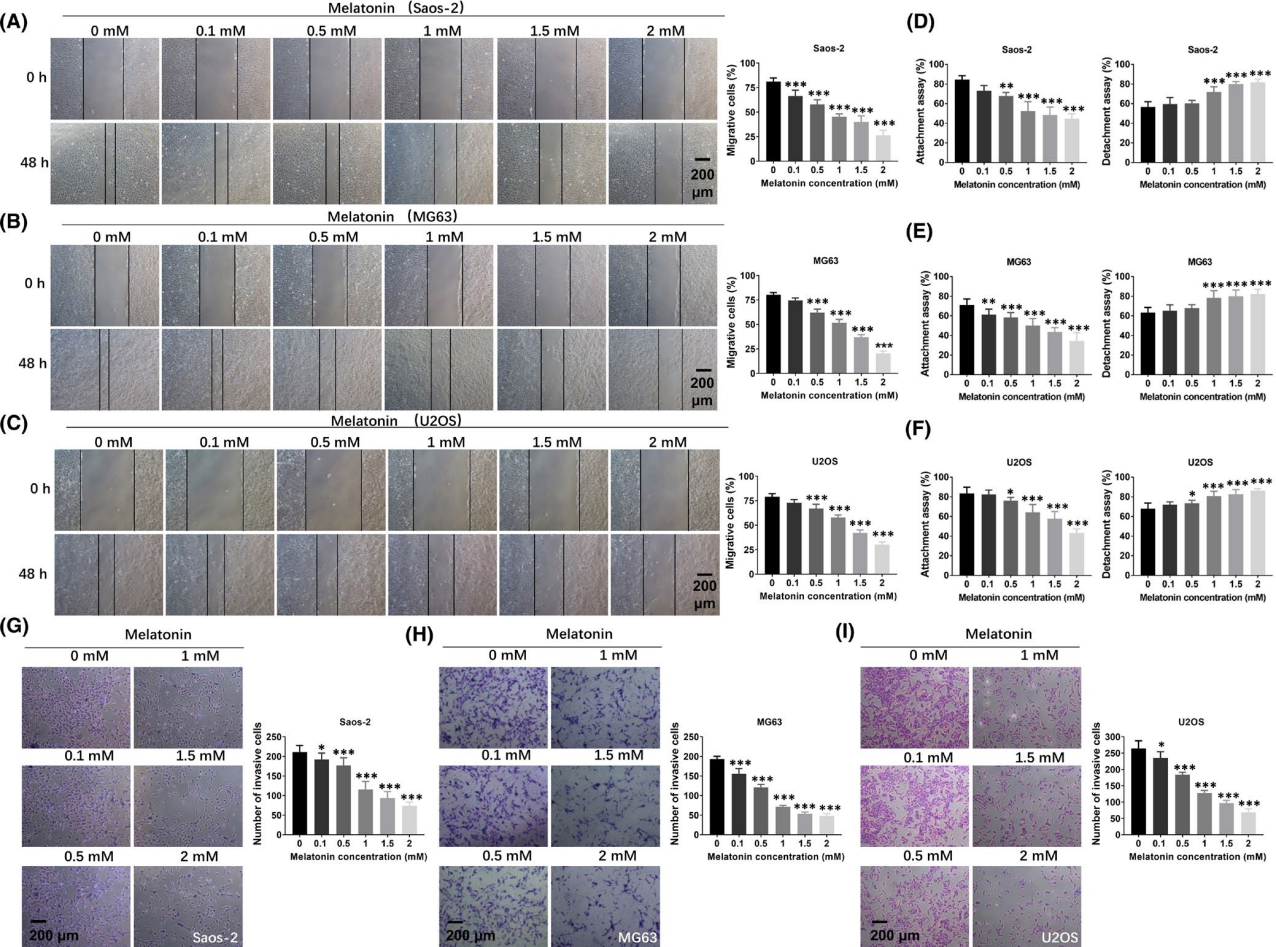
The influence of melatonin on the migration and invasion of OS cells. (A–C) The effects of melatonin on the migration ability of OS cells were detected by wound healing assay. (D–F) The functions of melatonin in the metastasis of OS cells were determined by attachment assay and detachment assay. (G‐I) The roles of melatonin in the cell invasion were assessed by Transwell assay. Scale bar = 200 μm. Significant difference relative to control group was presented as *p < 0.05; **p < 0.01 and ***p < 0.001

To investigate whether melatonin affected the invasive and metastatic ability of OS cells, three OS cell lines were treated with different concentrations of melatonin (0.1, 0.5, 1, 1.5 and 2 mM) for 48 h, and the wound healing assay, attachment/detachment assays and transwell assay were applied. As presented in Figure 2A–C, the results from the wound healing assay showed that, compared to the control group, the dose‐dependent decrease in migration was observed in Saos‐2, MG63 and U2OS cells treated with melatonin. Melatonin at 1, 1.5 and 2 mM exerted the most optimal effects on the migration and disrupted the migration ability of these three cell lines (Figure 2A–C). Furthermore, the attachment/detachment assays showed that the metastasis of OS cells was gradually decreased in the presence of melatonin at concentrations ranging from 0.1 to 2 mM. The most pronounced effect concentrations were 1, 1.5 and 2 mM (Figure 2D–F). In addition, transwell assay revealed that the invasion ability of three cell lines was reduced dose‐dependently after treatment with melatonin for 48 h (Figure 2G–I). Collectively, these results suggested that melatonin could suppress the metastasis of OS cells.

### 3.3 LncRNA JPX is upregulated in OS cells and suppressed by melatonin treatment

**FIGURE 3:**
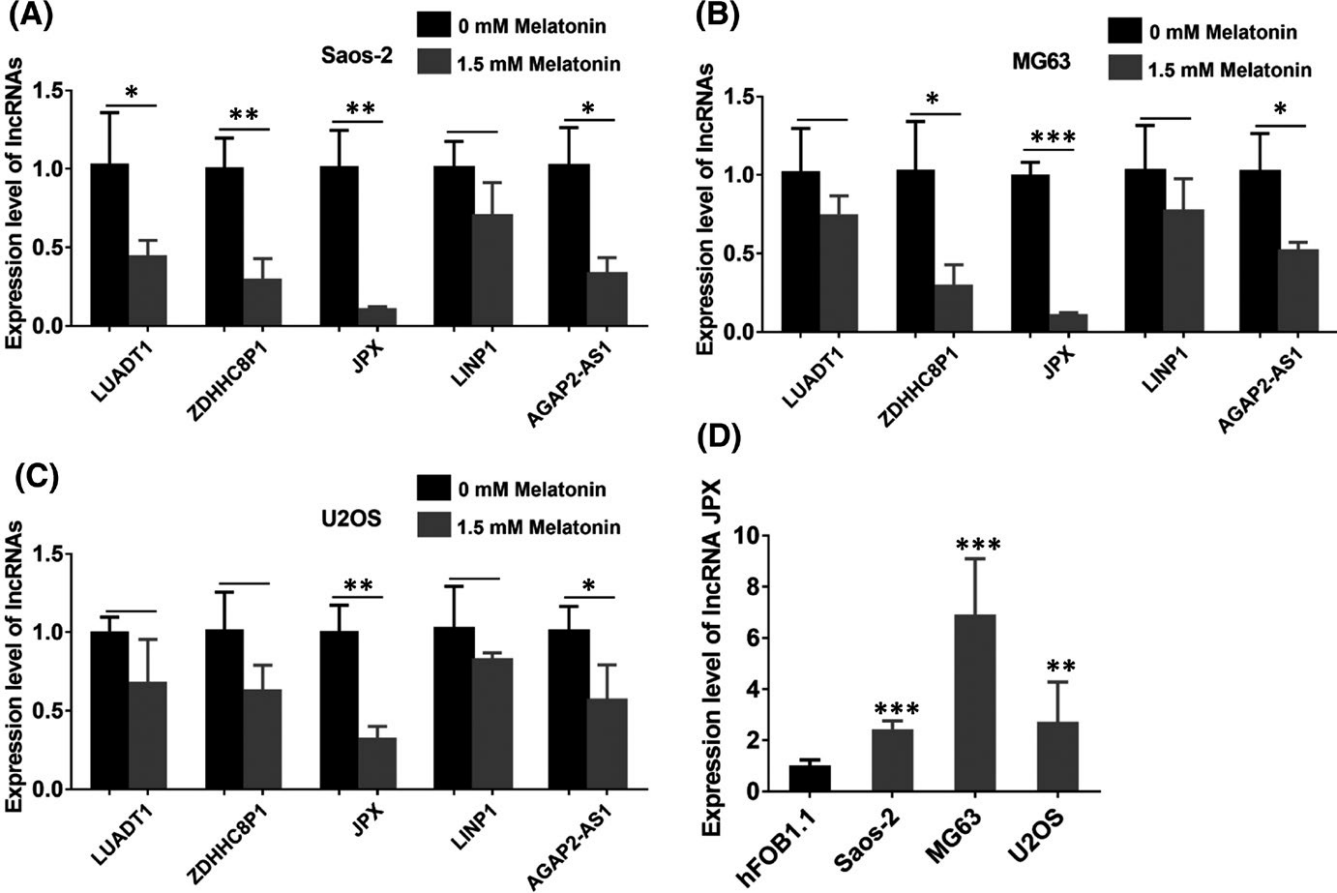
Expression level of lncRNA JPX in OS cell lines. (A‐C) The expression level of lncRNA LUADT1, ZDHHC8P1, JPX, LINP1, AGAP2‐AS1 was measured by real‐time qPCR analysis in Saos‐2 (A), MG63 (B) and U2OS (C) cells after treatment with 1.5 mM melatonin. (D) Expression level of lncRNA JPX in OS cell lines (Saos‐2, MG63 and U2OS) and human osteoblasts. Significant difference relative to control group was presented as *p < 0.05; **p < 0.01 and ***p < 0.001

It has been widely reported that lncRNAs participate in the development of many cancer types.^41^, ^42^, ^43^, ^44^ In order to determine whether lncRNAs were involved in the melatonin‐mediated anti‐cancer effects in OS cells, we performed real‐time qPCR analysis to identify differentially expressed lncRNAs in melatonin treated human OS cell lines. LncRNA LUADT1, ZDHHC8P1, JPX, LINP1 and AGAP2‐AS1 have been reported to participate in the development of cancers.^45^, ^46^, ^47^, ^48^, ^49^ However, the expression and roles of these lncRNAs in OS cells still remained unclear. Thus, we selected these lncRNAs (lncRNA LUADT1, ZDHHC8P1, JPX, LINP1, AGAP2‐AS1) as candidate genes in the subsequent experiments. Considering the inhibitory effects of melatonin on cell survival, cell viability, proliferation, migration and invasion, melatonin at 1.5 mM was selected in the further experiments. As shown in Figure 3A–C, we found that the expression of lncRNA JPX was the lowest expressed lncRNA in 1.5 mM melatonin treated OS cell lines. Thus, we selected lncRNA JPX as the potential target of melatonin in the further experiments. To further confirm the roles of lncRNA JPX, we measured the expression level of lncRNA JPX in human osteoblast cell line hFOB1.1 and human OS cell lines (Saos‐2, MG63 and U2OS). As displayed in the results of real‐time qPCR analysis, the level of lncRNA JPX was much higher in OS cells compared with human osteoblasts, which indicated that lncRNA JPX was upregulated in OS cells (Figure 3D). Thus, we hypothesized that lncRNA JPX might be related to the development of OS and participate in the melatonin‐mediated anti‐cancer effects in OS cells.

### 3.4 Overexpression of lncRNA JPX promotes the cell growth and metastasis of OS cells

**FIGURE 4:**
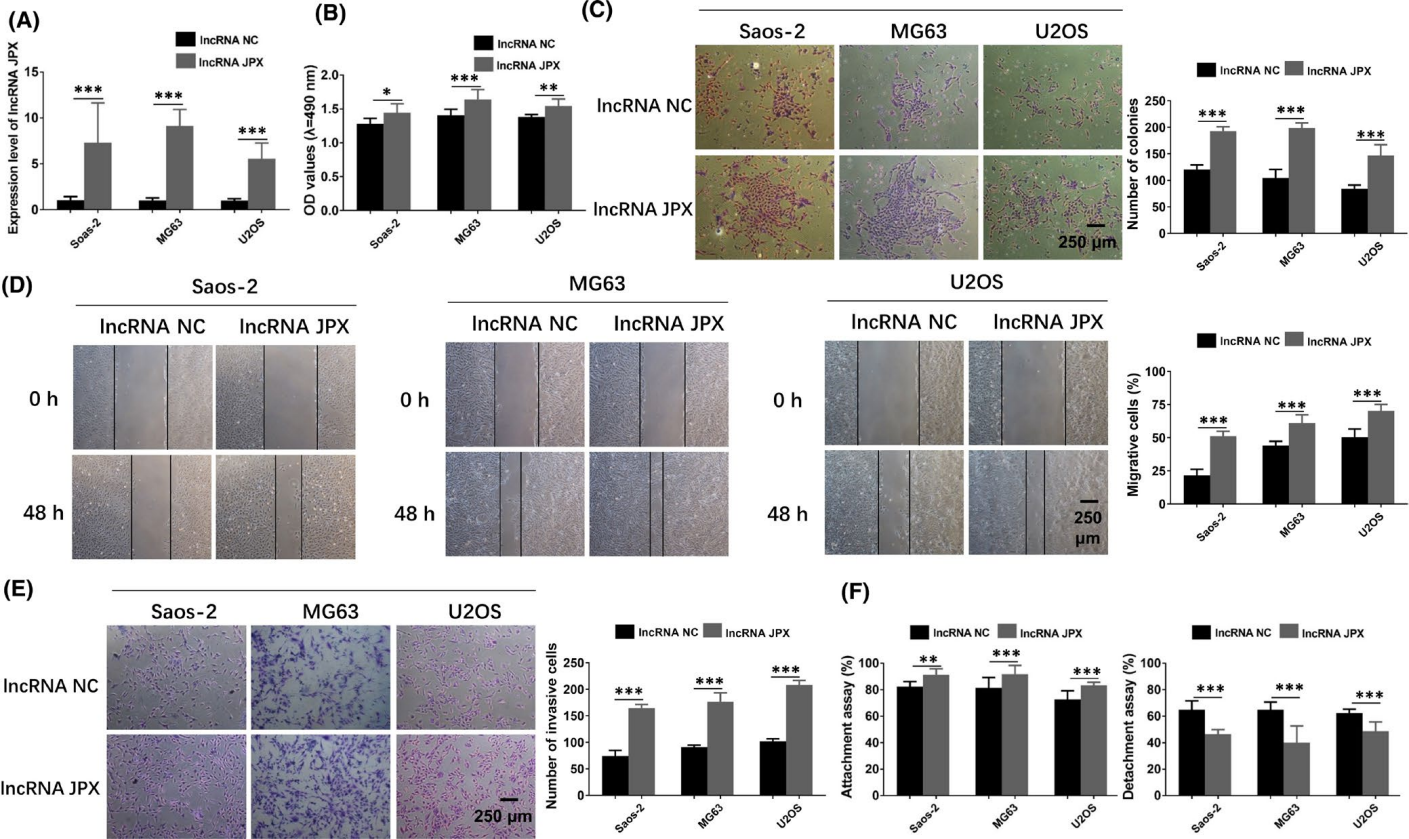
LncRNA JPX enhanced the proliferation and metastasis of OS cells. (A) The expression level of lncRNA JPX was examined in OS cells transfected with lncRNA JPX and lncRNA NC. (B) The cell viability of OS cells transfected with lncRNA JPX was evaluated by MTT assay. (C) The proliferative ability of OS cells treated with lncRNA NC and lncRNA JPX was examined by using colony formation assay. (D) The cell migration elevated by lncRNA JPX was detected with wound healing assay. (E) Transwell assay was conducted to detect the cell invasion ability of OS cells after transfected with lncRNA JPX. (F) Attachment assay and detachment assays were applied to analyse the effects of lncRNA JPX on the metastasis of OS cells. Scale bar, 250 μm. Significant difference relative to control group was presented as *p < 0.05; **p < 0.01 and ***p < 0.001

To investigate the roles of lncRNA JPX in OS cells, we investigated the effects of its gain or loss of function. First, lncRNA JPX was overexpressed in Saos‐2, MG63 and U2OS cells, and real‐time qPCR analysis was used to measure the expression of lncRNA JPX in OS cells after transfection. The results showed that the expression of lncRNA JPX in lncRNA JPX overexpression group was much higher compared with that in the negative control of lncRNA JPX (lncRNA NC) group in Saos‐2, MG63 and U2OS cells, indicating that lncRNA JPX was transfected into the OS cells successfully (Figure 4A). To explore the roles of lncRNA JPX in the cell viability of OS cells, OS cells were transfected with lncRNA JPX and lncRNA NC for 48 h. MTT assay showed that the OD values were elevated in the OS cells after lncRNA JPX transfection, suggesting that lncRNA JPX overexpression significantly improved the cell viability of OS cells (Figure 4B). As shown in the results of colony formation assay, overexpression of lncRNA JPX significantly promoted the proliferation of OS cells (Figure 4C). The results of the wound healing assay showed that the percentage of migrated cells in lncRNA JPX group was higher than that in lncRNA NC group, indicating that upregulation of lncRNA JPX favoured the migration of OS cells (Figure 4D). We next tested whether lncRNA JPX was also able to facilitate the cell invasion of OS cells. As expected, the percentage of invasive cells was significantly increased in lncRNA JPX overexpressed cells, which suggested that the increase of lncRNA JPX promoted the invasion of OS cells (Figure 4E). Besides, in Saos‐2, MG63 and U2OS cells, overexpression of lncRNA JPX greatly increased the metastasis of these OS cells compared with that in lncRNA NC group (Figure 4F). Collectively, these results indicated that lncRNA JPX played a positive role in the progression of OS cells.

### 3.5 Knockdown of lncRNA JPX negatively regulates the cell growth and metastasis of OS cells

**FIGURE 5:**
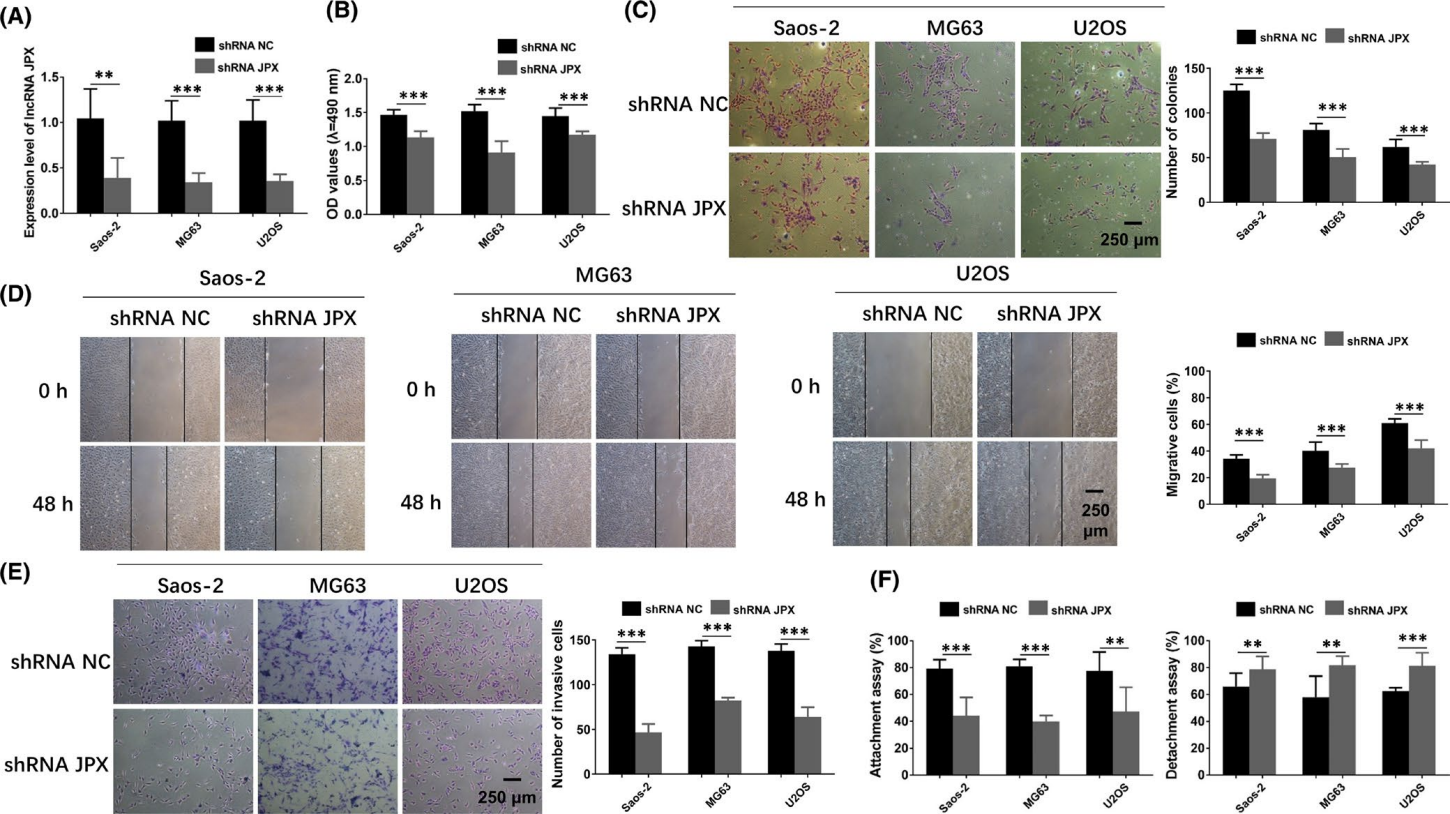
Knockdown of lncRNA JPX suppressed the proliferation and metastasis of OS cells. (A) LncRNA JPX was knockdown in OS cells by specific shRNA (shRNA JPX). The transfection efficiency was examined with real‐time qPCR analysis. (B) MTT assay was carried out to determine the cell viability in OS cells transfected with shRNA NC or shRNA JPX. (C) Colony formation assay was performed to test the proliferative ability of OS cells after knockdown of lncRNA JPX. (D) Wound healing assay was then utilized to assess the migration of OS cells after shRNA JPX transfection. (E) Invasion ability was examined in OS cells in shRNA JPX group by using transwell assay. (F) Attachment and detachment assays were conducted to detect the metastasis ability of OS cells transfected with shRNA JPX for 48 h. Scale bar, 250 μm. Significant difference relative to control group was presented as *p < 0.05; **p < 0.01 and ***p < 0.001

To test whether the reduction of lncRNA JPX inhibited the cell growth and metastasis of OS cells, we stably downregulated the level of lncRNA JPX in OS cells using shRNA JPX. Real‐time qPCR analysis revealed that lncRNA JPX was markedly decreased in shRNA JPX group compared with shRNA NC group in three human OS cell lines (Figure 5A). The results of MTT showed that the cell viability was obviously reduced in the shRNA JPX group compared with shRNA NC group in OS cells (Figure 5B). The results of colony formation assay demonstrated that cell proliferation was significantly restrained in the presence of shRNA JPX compared with shRNA NC treatment (Figure 5C). Besides, scratch assay displayed that the migration of OS cells was significantly inhibited after lncRNA JPX knockdown (Figure 5D). Furthermore, the decreased invasion ability of OS cells was also observed after knockdown of lncRNA JPX (Figure 5E). Furthermore, the knockdown of lncRNA JPX markedly reduced the metastasis of OS cells in comparison with the cells from shRNA NC group (Figure 5F). In short, the above data revealed that the low expression of lncRNA JPX effectively suppressed the cell growth and metastasis of OS cells.

### 3.6 Melatonin regulates the tumour growth and metastasis of OS cells by inhibiting the expression of lncRNA JPX

**FIGURE 6:**
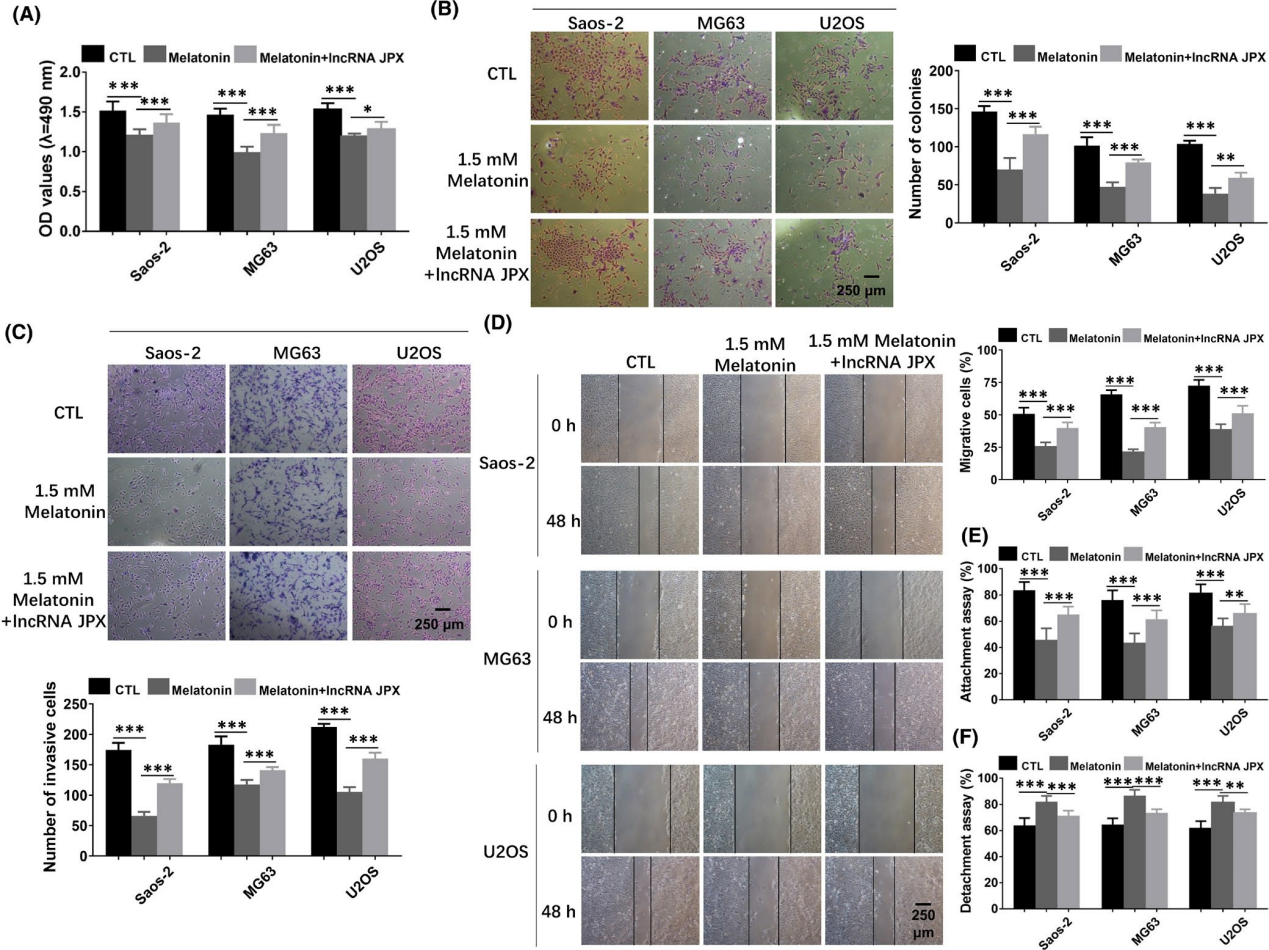
Melatonin inhibited the biological functions of OS cells by suppressing lncRNA JPX. (A) The reduced cell viability caused by melatonin treatment was reversed by overexpression of lncRNA JPX. (B) The decreased cell proliferation induced by melatonin was blocked by lncRNA JPX transfection. (C) Melatonin‐induced decrease in the invasion ability of OS cells was turned over in the presence of lncRNA JPX. (D) The attenuated migration of OS cells, which was caused by 1.5 mM melatonin, was partially reversed after lncRNA JPX transfection. (E and F) The attachment assay and detachment assays of OS cells in the presence of melatonin and lncRNA JPX. Scale bar = 250 μm. Significant difference relative to control group was presented as *p < 0.05; **p < 0.01 and ***p < 0.001

To further determine whether melatonin exerted its function through downregulation of lncRNA JPX, OS cells were treated with 1.5 mM melatonin or 1.5 mM melatonin+lncRNA JPX, respectively. As presented in Figure 6A, the functions of melatonin on the cell viability of OS cells could be reversed when the cells were co‐transfected with lncRNA JPX. Additionally, the results from colony formation assay showed that the inhibitory roles of melatonin in the proliferation of OS cells could be blocked by overexpression of lncRNA JPX (Figure 6B). As revealed in Figure 6C, the suppressive effects of melatonin on the invasion of OS cells were aborted by overexpression of lncRNA JPX. These findings reflected that melatonin could inhibit the cell growth and metastasis, and such function was mediated through suppressing the expression of lncRNA JPX. Wound healing assay indicated that the melatonin induced OS cell migration decrease, which was reversed after co‐transfection with lncRNA JPX (Figure 6D). The decreased percentage of attached cells in OS cells caused by melatonin was elevated by lncRNA JPX (Figure 6E). Besides, the promotive effects of melatonin in the detachment of OS cells were reversed after lncRNA JPX transfection (Figure 6F). The aforementioned data demonstrated that melatonin inhibited the OS growth and metastasis possibly through repressing the expression of lncRNA JPX.

### 3.7 Melatonin exerts its function through suppressing Wnt/β‐catenin pathway regulated by lncRNA JPX in OS cells

**FIGURE 7:**
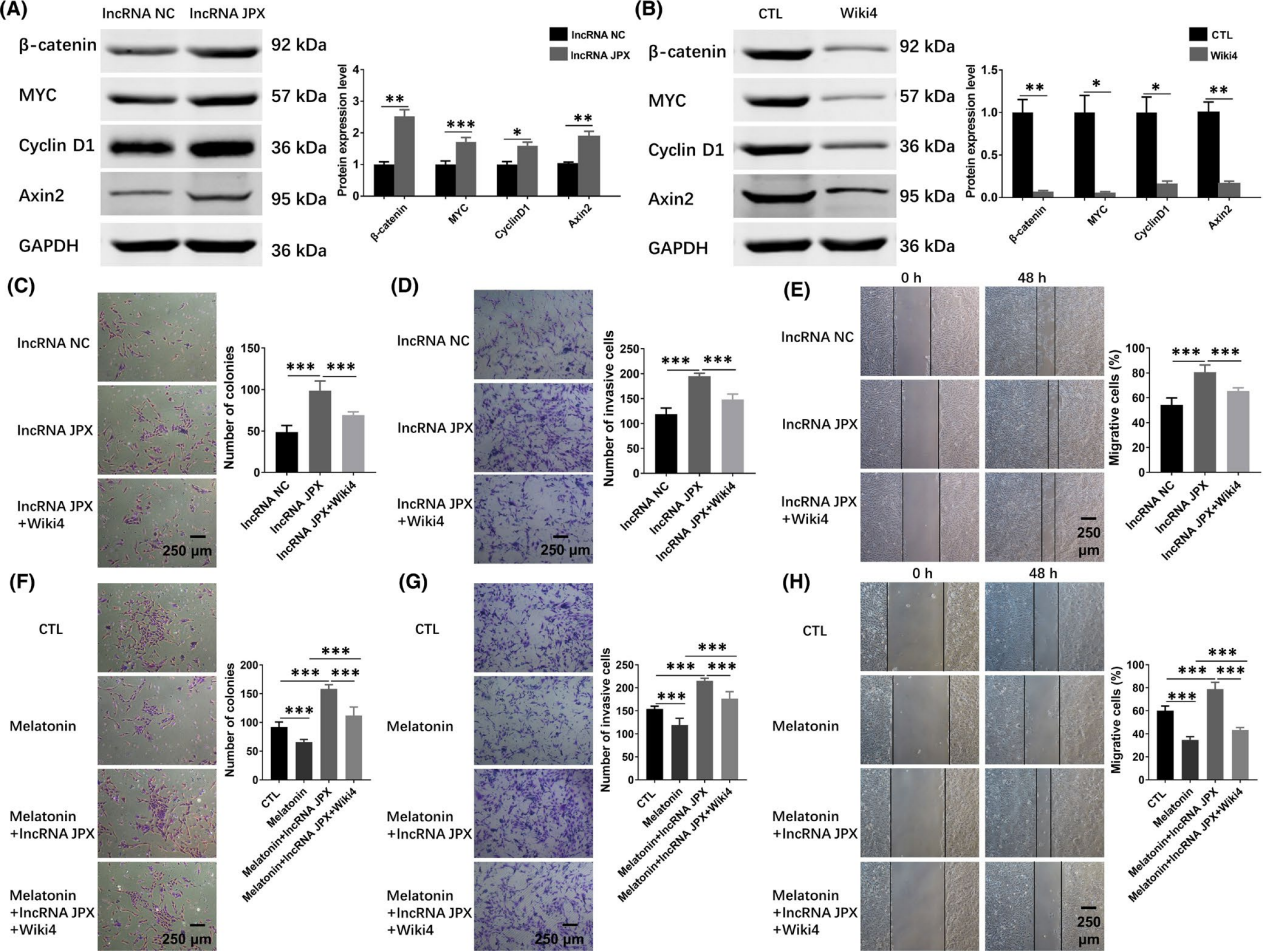
Wnt/β‐catenin signalling pathway was involved in lncRNA JPX mediated tumour progression in OS cells. (A) The protein levels of Wnt/β‐catenin signalling target genes were measured by western blot in MG63 cells transfected with lncRNA NC or lncRNA JPX. (B) The protein expression of the core factors of Wnt/β‐catenin signalling pathway was examined by western blot assay in MG63 cells treated with Wiki4. (C) The proliferative ability of MG63 cells treated with lncRNA JPX or lncRNA JPX + Wiki4 was examined with colony formation assay. (D) The invasion increased by lncRNA JPX was decreased in the presence of Wiki4. (E) The migratory ability of MG63 cells treated with lncRNA JPX or lncRNA JPX + Wiki4 was detected by wound healing assay (F‐H) The roles of Wnt/β‐catenin signalling in the inhibition of lncRNA JPX by melatonin. Scale bar = 250 μm. Significant difference relative to control group was presented as *p < 0.05; **p < 0.01 and ***p < 0.001

To uncover the potential mechanisms of lncRNA JPX regulating the biological behaviours of OS cells, we selected MG63 cells in the further analysis because the expression level of lncRNA JPX was the highest in MG63 cells among the three OS cells (Saos‐2, MG63 and U2OS). Considering that the Wnt/β‐catenin signalling pathway controls the important cellular events during cancer development and is important for cancer progression, we focussed on the Wnt/β‐catenin signalling pathway. To study whether lncRNA JPX regulated the biological functions of OS cells through the Wnt/β‐catenin pathway, we treated MG63 cells with lncRNA JPX for 48 h and detected the expression levels of key genes in the Wnt/β‐catenin signalling pathway. The results of western blot showed that overexpression of lncRNA JPX significantly increased the expression of β‐catenin, MYC, Axin2 and Cyclin D1 protein in MG63 cells, which indicated that the Wnt/β‐catenin pathway was activated by lncRNA JPX (Figure 7A). To further investigate whether the Wnt/β‐catenin pathway was involved in the lncRNA JPX mediated OS progression, Wnt/β‐catenin signalling was inactivated by using Wiki4, a widely used Wnt/β‐catenin pathway inhibitor. MG63 cells were treated with Wiki4 and co‐transfected with lncRNA JPX for 48 h. Western blot analysis indicated that, compared with the control group (lncRNA JPX group), the protein expression level of key genes in the Wnt/β‐catenin signalling pathway, including β‐catenin, MYC, Axin2 and Cyclin D1, was observably reduced by lncRNA JPX+Wiki4 treatment (Figure 7B). Using colony formation assay, we found that lncRNA JPX accelerated the proliferation of MG63 cells, but exhibited no influence on the proliferation ability in MG63 cells after Wiki4 treatment (Figure 7C). Accordingly, transwell assay showed that lncRNA JPX promoted the invasion of MG63 cells, but the action was abolished by Wiki4 treatment (Figure 7D). Furthermore, the wound healing assay also confirmed that lncRNA JPX accelerated the migration of MG63 cells, while this promotion was inhibited by Wnt/β‐catenin pathway inhibitor Wiki4 (Figure 7E). The above results indicated that lncRNA JPX participated in the tumour growth and metastasis through the Wnt/β‐catenin signalling pathway.

Furthermore, to clarify the role of Wnt/β‐catenin signalling in the inhibition of lncRNA JPX by melatonin, MG63 cells were treated with melatonin, melatonin + lncRNA JPX overexpression, melatonin + lncRNA JPX overexpression + Wiki4, respectively. Compared with the cells from the control group, melatonin markedly decreased the number of colonies, and overexpression of lncRNA JPX elevated the reduced number of colonies, which was caused by melatonin (Figure 7F). And this action was abolished in the presence of Wiki4 (Figure 7F). Besides, transwell assay suggested that overexpression of lncRNA JPX could increase the decreased number of invasive cells induced by melatonin treatment (Figure 7G). However, the treatment of Wiki4 could weaken this effect (Figure 7G). In addition, in Wiki4‐treated cells, overexpression of lncRNA JPX exerted no effects on the migration of MG63 cells, which was pretreated with melatonin (Figure 7H). Taken together, melatonin exerted its function by suppressing the expression of lncRNA JPX via regulating the Wnt/β‐catenin pathway in OS cells.

## 4 DISCUSSION

OS is a type of malignant tumour that begins in the BMSCs that form bones. Long bone is the most common site, like legs and arms. However, it could also start in any bones. Teenagers and young adults are the most affected population, but it could also affect younger children and older adults. The global incidence is about 1–3/million population.^50^ Surgery is usually performed to remove the tumour and metastasis, while chemotherapy is applied simultaneously. The limb amputation or salvage is usually performed according to the grade of OS. Although immunotherapy and pharmacogenomics therapies have been developed these years, the prognosis of patients with high‐grade OS is still poor. Thus, clarifying the underlying mechanism of OS development and developing effective treatment drugs are of great importance.

In recent years, the effects of natural products in tumour biology have been gradually concerned.^51^ Melatonin, a derivative of the amino acid tryptophan, is generally considered as a pleiotropic and multitasking molecule, which is mainly secreted by the pineal gland at night or in dark conditions.^52^, ^53^, ^54^ Numerous studies have shown that melatonin plays a critical and inhibitory effect on the pathogenesis of multiple types of tumours.^55^, ^56^, ^57^ For example, melatonin is used as a potential agent in the treatment and prevention of oral cancers through reducing oxidative stress.^58^ It has been reported that melatonin exerts anti‐proliferative actions via the activation of the MT1 receptor in breast cancer cells.^59^ Several studies have reported that melatonin exerts oncostatic effects in human OS cells.^60^, ^61^ However, the inhibitory effects of melatonin on OS and underlying mechanism have not yet been ascertained. In this study, we found that melatonin reduced the cell viability, proliferation, migration, invasion and metastasis of three OS cell lines, including Saos‐2, MG63 and U2OS, in a concentration‐dependent manner. Among 0.1, 0.5, 1, 1.5 and 2 mM, melatonin at 1, 1.5 and 2 mM exhibited the significant inhibitory effects on the biological functions of OS cells.

LncRNAs are a group of non‐coding RNA with more than 200 nucleotides in length.^62^, ^63^ LncRNAs could regulate the multiple processes, such as gene post‐transcriptional translation, chromatin remodelling and transcription.^64^, ^65^ LncRNAs have been proved to be involved in multi‐human cancers, including endometrial carcinoma, colorectal cancer, prostate, bladder and kidney cancer.^66^, ^67^, ^68^ In OS, lncRNAs have also been proved to regulate many processes and function as prognostic markers,^69^, ^70^, ^71^ therapeutic targets^72^, ^73^ and predictive markers.^74^, ^75^ However, the role of lncRNA JPX in OS has not been reported. In this study, the results showed that melatonin inhibited the expression of lncRNA JPX and the proliferation, migration and invasion of OS cells, which indicated that lncRNA JPX could be a key gene downstream of melatonin in OS cells. The promotion and inhibition of lncRNA JPX in OS cells confirmed its promotive roles in the development of OS. The anti‐tumour function of melatonin in OS cells was inhibited by overexpression of lncRNA JPX. These results indicated lncRNA JPX was the downstream key gene of melatonin in regulating the development of OS.

The Wnt/β‐catenin pathway could regulate multi‐tumour development, like lung cancer,^76^ intestinal tumour,^77^ gastric cancer^78^ and glioma.^79^ Evidence has proved that Wnt/β‐catenin pathway could regulate OS cell behaviours. The activation of the Wnt/β‐catenin pathway increases the chemoresistance of OS cells.^80^ Cinnamaldehyde promotes the cell apoptosis and inhibits the cell proliferation, migration and invasion in OS cells via inhibiting the Wnt/β‐catenin pathway.^81^ MiR‐1236‐3p inhibits the progression of OS via targeting Wnt3a 3’ UTR.^82^ Similarly, in this study, the Wnt/β‐catenin pathway was also inhibited by melatonin. This inhibitory role of melatonin in the Wnt/β‐catenin pathway was restricted by lncRNA JPX overexpression. Thus, the Wnt/β‐catenin pathway was the downstream pathway of the melatonin‐lncRNA JPX axis.

A variety of studies have reported that melatonin as a natural molecule plays anti‐cancer roles via the cause of epigenetic alterations, promotion of apoptosis, modulation of tumour metabolism and pro‐survival signalling, prevention of metastasis and angiogenesis. Studies conducted so far have not clearly shown the therapeutic potentials of melatonin in OS. In the present study, our results suggested that melatonin inhibited the biological functions of OS cells by repressing the expression of lncRNA JPX through regulating the Wnt/β‐catenin signalling pathway. We performed a series of experiments in vitro to detect the roles of melatonin in OS cells and its underlying mechanism. However, there were some limitations in this study. We did not perform in vivo experiments to assess the effects of melatonin on the tumour formation of OS cells. In the future, more experiments in vivo are needed to be conducted to detect the roles of melatonin in the tumour formation of OS cells. Besides, the benefits of melatonin in OS and whether melatonin administration to OS patients could extend the disease‐free survival are needed to be in‐depth investigated. Furthermore, analysis on the values of chemotherapy combined with melatonin in improving the efficacy and safety of cancer therapy is encouraged.

## 5 CONCLUSION

In this study, melatonin was proved to inhibit the proliferation, invasion and migration in a dose‐dependent manner of OS cells. LncRNA JPX‐Wnt/β‐catenin axis was confirmed to be the downstream pathway of melatonin inhibiting the biological behaviours of OS cells. Taken together, these data concluded that melatonin inhibited the biological functions of OS cells via regulating lncRNA JPX‐Wnt/β‐catenin signalling pathway. This study clarified the new molecular mechanism of OS and provided a novel treatment target in the following clinical investigation.

## CONFLICT OF INTEREST

No potential conflicts of interest were disclosed.

## AUTHOR CONTRIBUTION

**Yuan Li:** Conceptualization (equal); Data curation (equal); Formal analysis (lead); Funding acquisition (supporting); Project administration (lead); Supervision (equal); Visualization (equal); Writing‐original draft (equal); Writing‐review & editing (equal). **Jilong Zou:** Formal analysis (equal); Investigation (supporting); Project administration (supporting); Software (equal); Validation (equal). **Bo Li:** Conceptualization (equal); Investigation (supporting); Project administration (equal); Software (equal); Validation (equal); Writing‐review & editing (equal). **Jianyang Du:** Conceptualization (lead); Formal analysis (equal); Funding acquisition (lead); Investigation (equal); Project administration (equal); Validation (equal); Visualization (equal); Writing‐original draft (equal); Writing‐review & editing (equal).

## AUTHORS’ CONTRIBUTIONS

Yuan Li, Jilong Zou and Bo Li performed the experiments, analysed the data and wrote the manuscript. Jianyang Du and Bo Li initiated and organized the study. Jianyang Du, Yuan Li and Bo Li revised the manuscript.
